# Notes of Surgical Cases

**Published:** 1862-09

**Authors:** J. F. Miner


ART. III.—*Notes of Surgical Cases. By* J. F. MINER, M. D.

A brief history of a few cases of Strabismus recently operated upon, and the results of these operations, may be of interest, as showing their safety and propriety. They will also serve to illustrate some of the important points which are to be considered in connection with this subject.

*Henry P.*, aged 24 years, convergent strabismus of right eye from birth, or early infancy. The deviation was very great, and the affected eye was amblyopic.

February 16, gave chloroform, and divided tendon of the rectus internus.

May 16, deviation slight, yet still perceptable. Divide tendon and sub-conjunctival fascia without aid, or rather embarrassment from chloroform. The chloroform produced at the first operation great excitement, and added to the difficulties of the operation. The eyes rolled in all directions, and to judge of the effects of dividing the tendon was impossible.

Results of the second operation perfect in restoring the proper axis; deformity is wholly removed; power of vision is not affected to any great degree.

*Jane H.*, aged 8 years; convergent strabismus left eye, which is hereditary. Deviation very great; the cornea hid by the inner canthus, especially when the child is excited or embarrassed.

December 20, gave chloroform and divided the tendon of the rectus internus, and the fascia, making dissection upon the inner fourth of the eye, freeing the globe from all attachments, yet without uncommon division of conjunctiva. All deformity removed; the inner canthus undisturbed. Amount of improvement in vision unknown, since it was hardly ascertained previous to operation. At present, can read, with difficulty, words in large type, when the right eye is covered.

*Edward H.*, aged 7 years. Hereditary convergent strabismus right eye; the left eye also slightly convergent.

November 30, 1861, gave chloroform and divided the tendon of the rectus internus. The result seemed satisfactory for a few months, but at length the deformity still remained, in part at least.

June 10, 1862, gave sulphuric ether to complete anæsthesia, and again divided the tendon and sub-conjunctival fascia.

Result exceedingly satisfactory; all deformity in the eye operated upon removed, and the other eye also seems now to have assumed a straight position. Vision is defective, but may, and probably will, improve.

July 1st, removed a small fungus from the wound made in the conjunctiva, and since then there has been no trouble.

*Patrick B.*, aged 30 years; divergent strabismus of both eyes; much the greatest of left eye, which is amblyopic.

December 5th, divide the tendon of the rectus externus and the sub-conjunctival fascia. The globe assumed position to correspond exactly with the opposite eye, and is so satisfactory in its results that the patient desires no farther improvement. Increase of vision claimed by the patient, but has never been tested or demonstrated.

*William S.*, aged 42 years; divergent strabismus of right eye, with incipient cataract in both eyes. Had been operated upon for strabismus by a distinguished surgeon in February, 1861, without changing the axis of the eye.

February 10, 1862, preparatory to an operation for cataract, the tendon of rectus externus was divided, and also the sub-conjunctival fascia.

Results of this operation perfect; the strabismus being fully and perfectly cured. The squint in this case was supposed by the patient to have been caused by the disease of the internal eye. The eyes had been straight and healthy until the last three or four years.

*Charles W.*, of Colden, aged 24 years; convergent strabismus of left eye. The convergence greater than usual; vision imperfect; said to have been turned by convulsions, when a child.

March 28, divided the tendon and fascia. Results as to improvement of vision not known; relief of deformity complete.

*Simeon S*, aged 24 years; convergent strabismus of both eyes, produced by violent concussion of the brain or injury to the nerves, supplying the muscles of the eye.

January 14, 1862, divided the tendons of the internal recti-muscles, when proper axis was restored to the right eye, the left being paralyzed in its motions, and but slightly benefited.

March 18, the division of the tendon of the internal rectus of left eye was again made. The power of rotating the eye was still imperfect, and the results of the operation seemed uncertain.

July 14, 1862, examine the eyes, and find perfect restoration of sight, motion and axis.

The injury producing this turning of the eyes was received November 5 1861, so that it had existed but two months; not sufficiently long to injure the sight by disuse, and consequently the relief of deformity constituted a perfect cure.

*James C.*, aged 16 years. Hereditary convergent strabismus of right eye, with amblyopia.

December 30, 1861, divided the tendon of the rectus internus and sub-conjunctival fascia.

July 12, 1861, the eye perfectly in axis, and the power of vision increased. Result in all respects highly satisfactory.

*Mary S.*, of Gowanda, aged 18 years, convergent strabismus of left eye from earliest infancy, perhaps from birth. The right eye is also a very little inclined internally; left eye amblyopic.

May 8, 1862, divide the tendon and fascia in the usual manner, and the eye immediately assumes proper position.

May 11, find great effusion of blood between the conjunctiva and sclerotica, producing ecchymosis in marked degree; no inflammation or other unpleasant effects.

May 18. Perfect relief of deformity; power of vision as yet unchanged.

*Joseph C.*, aged 11 years, has convergent strabismus, which is probably hereditary. Other members of the family have the same deformity.

July 7, 1862, make the usual operation for strabismus, and obtain perfectly satisfactory results.

The young lad has been out considerably in the streets and storms, and has suffered from inflammation more than is common when proper care is taken to prevent it, yet not in any degree requirng treatment to subdue it.

*Timothy J. K.*, aged 20, has divergent strabismus and amblyopia.

April 26th, divided the tendon of the rectus externus of the left eye, when it immediately assumed position in axis with the other.

July 12, vision unchanged; can see to do fine work better, if the eye operated upon is closed.

*James F.*, has spasmodic strabismus, or constant uncontrollable rotation, the globes turning inward with rapid motion; has myopia and amblyopia. Divide the tendon of the rectus internus of the right eye, and a few months later, the left eye. These operations were attended by great improvement in the steadiness of the eye. Vision is unaffected, other than arises from ability to fix the eye more steadily.

There is no operation in surgery in itself so simple and attended by so little risk of unpleasant consequences, and by so little pain, which relieves so great a deformity, and at the same time affords some prospect of improvement in vision as the operation for strabismus. In most cases of long standing, vision in the eye most affected will be found defective, but rarely will it be wholly destroyed, This amblyopia is more often produced by the disuse of the eye than by other cause; inability to bring the eye into proper axis, having led to the expedient of turning it wholly away, or to disregard by the nerve and brain of any impression made upon the retina.

Eyes that have been turned for a long time do not again regain the power of vision, only to a limited extent, and in many instances no improvement can be discovered. This is equally true of strabismus from all causes and not much variation has been observed in this respect whatever might have been the injury or influence which produced it.

Children obtain better vision than adults, and cases early restored when turned by injuries of the brain or nerves supplying the muscles, are much more likely to be satisfactory in this respect, than cases which are long delayed.

In favorable cases much is to be hoped in improvement of vision, but this should not be over-estimated; vision will rarely be fully regained, and in some cases of long standing, no improvement whatever will be produced.

Relief from deformity will, in almost all cases, be obtained; this it is safe and proper to predict, and we shall rarely if ever be disappointed, unless we mistake immoveable distortion from disease of the brain or paralysis of the muscles, for strabismus. When the motions of the eye remain so that there is considerable freedom when the opposite eye is covered, we may safely conclude that a proper operation will be followed by satisfactory results.

It is not proposed to say much about the manner of operating, or the the kind of operation most desirable, but a remark or two will be excused.

The tendon being divided, the eye does not always resume its proper position; the fascia will often require division to considerable extent and it is not uncommon to be obliged to dissect it from its attachments to the globe for a distance of nearly or quite one-fourth its circumference before satisfactory result is obtained. Failure from want of observance of this, is common, and we have only one rule in operating, in cases where the eye does not appear straight, and that is, to divide not only the tendon but the sub-conjunctival fascia until the eye is seen to have assumed a proper position, and by following this plan we have succeeded, in several instances, in obtaining perfectly satisfactory results, after failure by the most distinguished and experienced operators.

To prevent deformity from retraction of the caruncle, it is only necessary to make the section of the conjunctive a little nearer the cornea, than is often practiced when the section may be carried to any desired extent and not the slightest deformity will generally be produced. If the caruncle should be found to retract the deformity is only observable by professional eyes, and can hardly be called a deformity when compared with strabismus.

